# Twist promotes angiogenesis in pancreatic cancer by targeting miR-497/VEGFA axis

**DOI:** 10.18632/oncotarget.8269

**Published:** 2016-03-22

**Authors:** An Liu, Chenggang Huang, Xuehong Cai, Jia Xu, Dinghua Yang

**Affiliations:** ^1^ Department of Hepatobiliary Surgery, NanFang Hospital of Southern Medical University, Guangzhou, 510515 Guangdong, China; ^2^ Department of General Surgery, The First People Hospital of Yueyang, Yueyang, 414000 Hunan, China

**Keywords:** microRNA, tumor biology, proliferation, metastasis

## Abstract

Angiogenesis is a critical step in the growth and dissemination of malignant diseases, including pancreatic cancer. Twist has been shown to stimulate angiogenesis in the tumor site. However, whether Twist contributes to angiogenesis in pancreatic cancer remains unknown. In this paper, we found that the expression of Twist was significantly increased in human pancreatic cancer cell lines and pancreatic cancer specimens. It is also closely engaged to adverse clinical feature, diminished survival and angiogenesis in pancreatic cancer patients. The up-regulation of Twist was found to be promoting cell growth, invasion and tubule formation of human umbilical vein endothelial cells (HUVECs) in *vitro*. By contrast, the silencing of Twist inhibited orthotopic xenograft tumor growth, metastasis and angiogenesis. Subsequent investigations disclosed that Twist was regulated by miR-497 directly, leading to the increased level of Vascular Endothelial Growth Factor-A (VEGFA). Moreover, gain-of-function and loss-of-function studies demonstrated that miR-497 could suppress the pro-proliferative, angiogenic and metastatic ability of pancreatic cancer cells. The ectopic expression of VEGFA obviously abrogated the anti-angiogenic effect induced by Twist knockdown, whereas the silencing of VEGFA markedly rescued the pro-angiogenic effect of Twist. By analyzing the expression levels of miR-497, Twist was found inversely correlated with miR-497 in pancreatic cancer tissues, and a positive correlation was found between Twist and VEGFA levels in pancreatic cancer specimens. In conclusion, our results suggested that the Twist/miR-497/VEGFA axis is significantly correlated with metastasis and angiogenesis in pancreatic cancer.

## INTRODUCTION

Pancreatic cancer is the 7^th^ most frequent cause of cancer death worldwide, with approximately 43,920 people in the United States have suffered from pancreatic cancer, and causing 37,390 people died from pancreatic cancer in 2012 [1, 2]. As a highly aggressive malignant disease, survival at advanced stages of pancreatic cancer is poor. The 5-year survival rate of patients with pancreatic cancer is the lowest amongst all solid cancers, with a median survival of 6 months [3]. During the last two decades, despite the availability of myriad of treatment modalities, the 5-year overall survival for pancreatic cancer only slightly improved [4]. The identification of potential biomarkers for the prediction of recurrence, disease-free survival and overall survival remains a priority. Nowadays, along with the advance in the study of the cellular immunology and molecular etiology of pancreatic cancer, novel therapeutic strategies, such as immunological therapy and genetic treatment, have emerged [5, 6].

Angiogenesis is the formation of new capillary blood vessels under physiological and pathological conditions, and is vital for tumorigenesis and metastasis [7, 8]. Nowadays, targeting tumor angiogenesis is regarded as a well-established strategy for cancer treatment. Numerous anti-angiogenic agents have been applied in pancreatic cancer clinically with limited survival benefit [9-11]. Moreover, recent studies demonstrated that the strategy of anti-angiogenic could inhibit primary tumor growth but can promote metastasis in animal xenograft tumors, including pancreatic cancer [12, 13]. Therefore, further investigations on anti-angiogenic attempts in pancreatic cancer should include agents that can combat metastasis as well as blood vessel growth.

In embryonic development, Twist is an essential transcription factor for cell migrating and tissue reorganising properly [14]. It has been suggested that Twist is responsible for metastasis, cisplatin chemoresistance and epithelial to mesenchymal transition in pancreatic cancer [15, 16]. It also increases miRNA-10b, which is positively related to cell migration, invasion, and metastasis. Recent study demonstrated that the up-regulation of Twist contributed to tumor angiogenesis [17]. Therefore, Twist may be a novel therapeutic target for tumor metastasis and angiogenesis. As a member of VEGF family, VEGFA is frequently over-expressed in various human solid tumors [18]. The association of VEGF and cancer is well documented [19, 20]. However, the relationship between Twist and VEGFA in the development of solid cancer has not been fully delineated or appreciated.

Nowadays, although few authors have arguably showed that miR-497 exert proliferative role in malignant neoplasms [21], but the weight of evidence favors the anti-proliferative role of miR-497 [22-24]. A previous study demonstrated that miR-497 induced cell apoptosis by negatively regulating Bcl-2 protein expression at the post-transcriptional level in human breast cancer [22]. Recent studies found that miR-497 blocks liver cancer cell cycle at G1 phase by suppressing the expression of CDK4, CCNE1 and BTRC [24]. However, whether the dysregulation of miR-497 contributes to pancreatic cancer metastasis and angiogenesis is currently unclear. In this study, for further investigate the interrelationship between Twist, miR-497 and VEGFA in pancreatic cancer, we examined the effect of Twist/miR-497/VEGFA axis in metastasis and angiogenesis in pancreatic cancer cells both in *vitro* and in *vivo*.

## RESULTS

### Twist is over-expressed and correlated with pancreatic cancer progression

**Figure 1:**
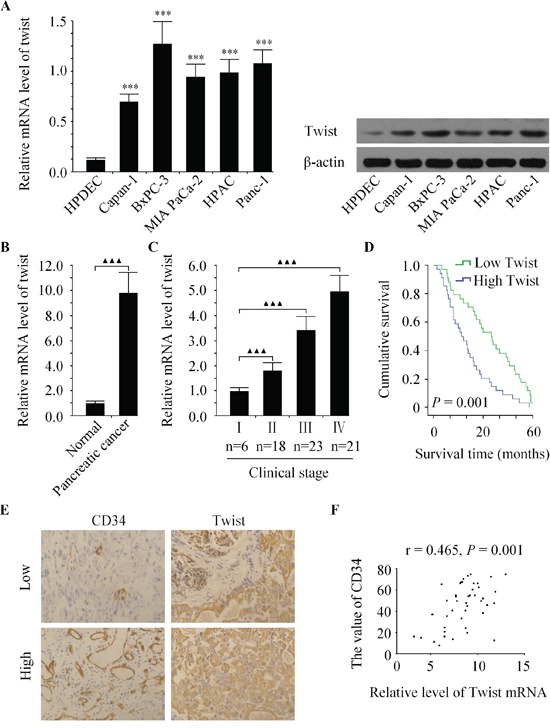
Twist is over-expressed and correlated with pancreatic cancer progression **A.** The mRNA and protein expression of Twist were increased in five human pancreatic cancer cell lines compared to non-malignant pancreatic epithelial cell line HPDEC. **B.** Relative Twist expression was increased in pancreatic cancer tissues compared with the matched adjacent noncancerous tissues. **C.** Relative expression of Twist was positively correlated with clinical stages of pancreatic cancer. **D.** Kaplan-Meier curve of overall survival for patients whose pancreatic tumors expressed high or low levels of Twist. **E.** Representative images immunostained with antibody recognizing CD34 with low or high expression of Twist. **F.** Twist mRNA expression was positively correlated with MVD in pancreatic cancer tissues. ***P < 0.001 vs. HPDEC group, ^▲▲^*P* < 0.01, ^▲▲▲^*P* < 0.001.

We first examined the relative expression of Twist in several pancreatic cancer cell lines, namely Bxpc-3, MIA PaCa-2, Capan-1, Panc-1 and HPAC, along with HPDEC, which is a human non-malignant pancreatic duct epithelial cell line, by qRT-PCR and western bolt. The results showed that in all the five pancreatic cancer cell lines, the level of Twist was found higher than in the HPDEC (Figure 1A). Next, we evaluated the expression of Twist in 68 pairs of human pancreatic cancer clinical samples and their corresponding normal pancreatic tissues. Consistently, Twist was also increased in pancreatic cancer tissues in comparison with normal tissues (Figure 1B). We further investigated the clinical relevance of Twist in pancreatic cancer progression. The data showed that Twist expression was positively correlated with clinical stage in pancreatic cancer patients (Figure 1C). Statistical analysis revealed that the patients with lower Twist level had a longer survival time than those with higher Twist expression (Figure 1D). Moreover, we found that tumors with enhanced Twist expression had markedly greater MVD (Figure 1E), and the abundance of Twist was positively correlated with that of MVD in pancreatic cancer tissues (Figure 1F). Our data demonstrated that the expression of Twist was strongly associated with tumor angiogenesis in pancreatic cancer.

### Effects of twist on the cell growth, invasion and tubule formation of HUVECs in *vitro*

**Figure 2:**
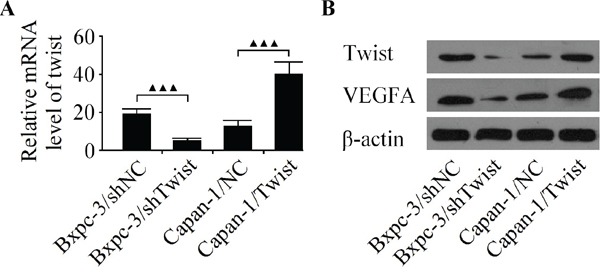
Analyze the expression of Twist and VEGFA in Bxpc-3 and Capan-1 cell lines **A.** The expression of Twist mRNA was detected by qRT-PCR in pancreatic cancer cells infected with the LV-shTwist, LV-Twist or their matched negative controls. **B.** Western blot analysis of Twist and VEGFA in Bxpc-3 and Capan-1 cells after infection. Results are reported as the mean ± SD of four independent experiments, ^▲▲▲^*P* < 0.001.

To elucidated whether or not Twist could facilitate angiogenesis in pancreatic cancer cells, the Bxpc-3 cell line with high metastatic potential and the Capan-1 cell line with low metastatic potential were used in the subsequent experiments [26]. As shown in Figure 2, after infecting pancreatic cancer cells with LV-Twist, LV-shTwist or negative controls, the expression of Twist was explored by qRT-PCR and western blot. As a result, infection of LV-Twist let to a significant increase in its expression. Moreover, the Twist level was decreased markedly after the Bxpc-3 cells were infected with LV-shTwist. Furthermore, western blot analysis showed that the decreased expression of Twist reduced the level of VEGFA in Bxpc-3 cells, whereas the level of VEGFA was increased after Capan-1 cells were infected with LV-Twist (Figure 2B).

**Figure 3:**
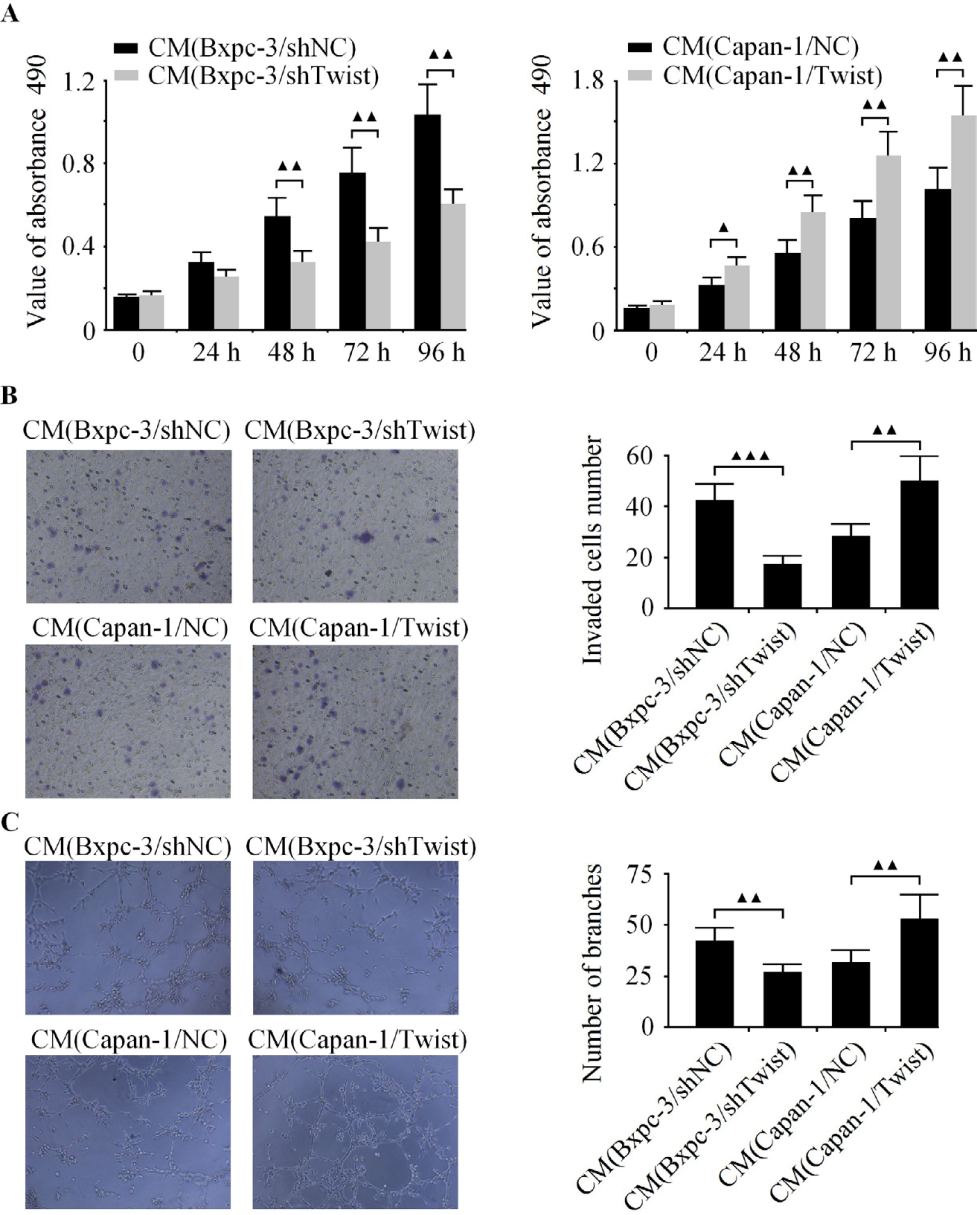
Effects of Twist on the cell growth, invasion and tubule formation of HUVECs in *vitro* Bxpc-3 cells were infected with LV-shTwist to decrease the level of Twist, and Capan-1 cells were infected with LV-Twist to increase the expression of Twist. Then the media were collected as CM and applied to HUVECs. **A.** The cell growth of HUVECs was analyzed by CCK-8 assay. **B.** The cell invasion was examined by boyden chamber assay. **C.** The tubule formation of HUVECs was measured by capillary tube formation assay. The results are representative of three independent experiments, and the values are presented as the mean ± SD, ^▲▲▲^*P* < 0.001, ^▲▲^*P* < 0.01, ^▲^*P* < 0.05.

Next, the CCK-8, boyden chamber and capillary tube formation assay were used to evaluate the angiogenic activity of HUVECs. It was observed that the CM from Twist-depleted Bxpc-3 cells inhibited cell growth, invasion and tubule formation of endothelial cells (Figure 3A-3C). In contrast, the CM from Twist-upregulated Capan-1 cells promoted cell growth, invasion and tubule formation of HUVECs (Figure 3A-3C).

### The down-regulation of twist represses pancreatic cancer growth, metastasis and angiogenesis in *vivo*

**Figure 4:**
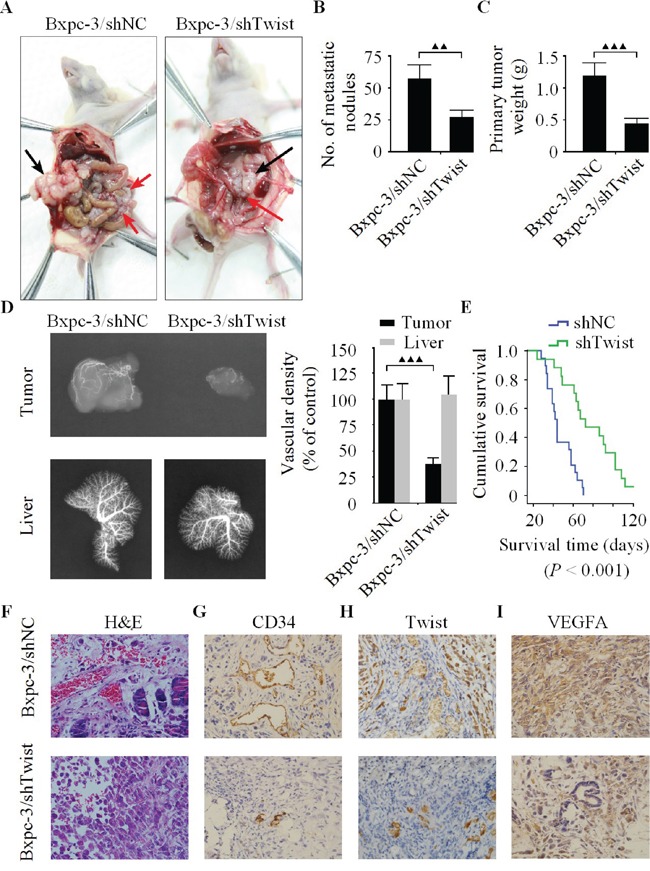
The down-regulation of Twist represses pancreatic cancer cell growth, metastasis and angiogenesis in *vivo* **A.** Necropsy photographs of nude mice bearing pancreatic cancer showing therapeutic benefit of shTwist. The black arrows show the primary tumors, and the red arrows show the metastatic nodules. **B.** Number of metastatic nodules in the Bxpc-3/shTwist group was markedly less than Bxpc-3/shNC group. **C.** The primary tumor weights showed significant decrease in the Bxpc-3/shTwist group compared with Bxpc-3/shNC group. **D.** The vascular images of the tumors and livers were captured by X-ray machine after lead oxide-gelatin injection. **E.** The survival rate of the mice from Bxpc-3/shTwist group was significantly better than those of the Bxpc-3/shNC group (n = 19 for each group). H&E **F.** and immunohistochemistry for CD34 **G.**, Twist **H.** and VEGFA **I.** protein expression in harvested tumors from tumor bearing nude mice (original magnification, ×400). Immunoreactivity towards all proteins were reduced in the Bxpc-3/shTwist group relative to their expression levels in the Bxpc-3/shNC group. Similar results were obtained in three independent experiments, ^▲▲▲^*P* < 0.001.

On account of the important role of Twist in pancreatic cancer, to indentify the pro-angiogenic effect of Twist in *vivo*, orthotopic xenograft tumor model was performed to compare the tumorigenesis of Bxpc-3/LV-shNC (Bxpc-3/shNC) and Bxpc-3/LV-shTwist (Bxpc-3/shTwist) cells. At the termination of experiment, the primary pancreatic tumor were excised neatly and the macroscopic nodules of peritoneal dissemination were counted. As shown in Figure 4A, metastatic nodules developed preferentially around the vessels of the intestinal mesentery. The total number of metastatic nodules was 56.3 ± 10.6 in the Bxpc-3/shNC group, whereas the Twist knockdown (Bxpc-3/shTwist) group showed comparatively fewer nodules (26.7 ± 4.4, *P* < 0.01) (Figure 4B). The primary tumor weights were indicative of a significant decrease in the Bxpc-3/shTwist group compared with Bxpc-3/shNC group (Figure 4C). Furthermore, tumor vascular imaging analysis revealed that the vascular density in the Bxpc-3/shTwist group was markedly reduced in comparison with Bxpc-3/shNC group (Figure 4D). To rule out the possibility of non-even perfusion of lead oxide injectate in the vasculature. We evaluated the vascular density of liver in both groups. Our results demonstrated that the vascular density of liver in Bxpc-3/shTwist group was not as statistically significant as the Bxpc-3/shNC group (Figure 4D, right). It was proved that our perfusions of lead oxide injected in the vascular systems of both groups of animals were performed evenly. In addition, the survival curve of these mice is shown in Figure 4E. Consistent with the finding of macroscopic peritoneal metastasis, survival analysis showed that the median survival was significantly prolonged in the Bxpc-3/shTwist group versus Bxpc-3/shNC group. Taken together, such outcomes indicated that silencing of Twist can effectively inhibit the metastasis and angiogenesis as well as improve the prognosis of pancreatic cancer in this experimental condition.

### Tumor immunohistochemistry in *vivo*

H&E evaluation of the tumors from both groups showed high grade carcinoma associated with tumor apoptosis and necrosis. However, there were differences in the vascular density and pattern of necrosis among the two groups. The tumors from Bxpc-3/shNC group are characterized by a abundant arteriolar supply with severe morphologic abnormalities such as contour irregularities, tortuous and elongated vessel segments, while the necrosis was majorly observed in the central area, whereas the peripheral tumor was largely viable and consisted of large nests of neoplastic cells with minimal intratumoral stroma (Figure 4F, upper row). In contrast, the blood supply in the Bxpc-3/shTwist group was relatively reduced, and large areas of cell debris due to marked tumor destruction in the tissue sections were seen (Figure 4F, lower row). Moreover, the MVD was noticeably reduced in the Bxpc-3/shTwist group in comparison with Bxpc-3/shNC group (Figure 4G). Subsequently, tumors with low Twist level in Bxpc-3/shTwist group had significantly lesser VEGFA expression than Bxpc-3/shNC group (Figure 4H-4I). Overall, these results suggested that Twist was directly involved in the promotion of angiogenesis as well as tumor metastasis in nude mice.

### Twist is a direct target of miR-497

**Figure 5:**
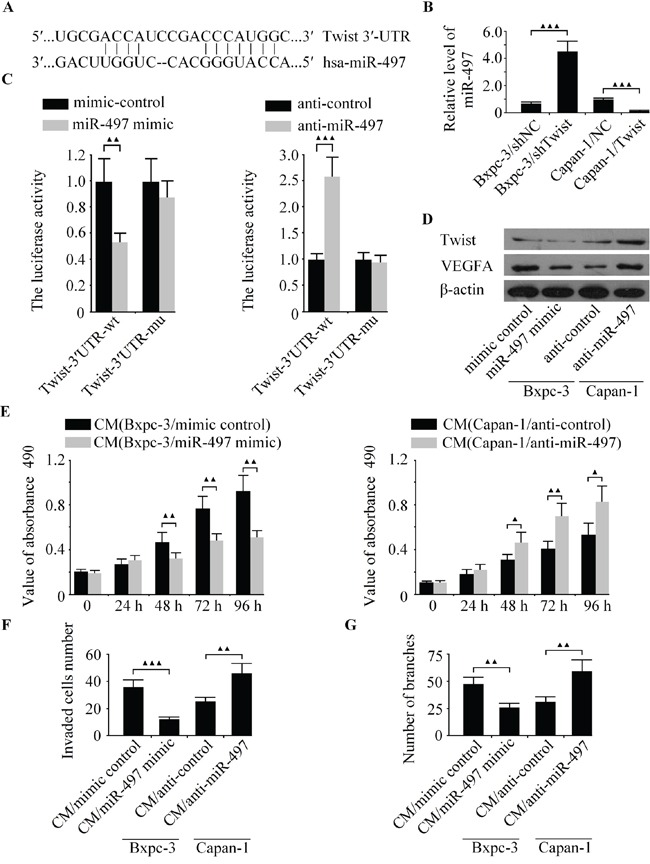
The expression of Twist was regulated by miR-497 directly **A.** The miR-497 binding site in the 3′-UTR of Twist mRNA. **B.** The level of miR-497 was detected in Bxpc-3 and Capan-1 cells by qRT-PCR. **C.** The relative luciferase activity was measured in Bxpc-3 cells after co-transfection of Twist-3′UTR-wt or Twist-3′UTR-mu with miR-497 mimic, anti-miR-497 or appropriate controls. **D.** The protein levels of Twist and VEGFA was examined in Bxpc-3 and Capan-1 cells by western blot. Bxpc-3 cells were transfected with miR-497 mimic to increase the expression of miR-497, and Capan-1 cells were transfected with anti-miR-497 to decrease the level of miR-497. Then the media were collected as CM and applied to HUVECs. **E.** The cell growth capacity of HUVECs was analyzed by CCK-8 assay. **F.** The cell invasion was examined by boyden chamber assay. **G.** The tubule formation of HUVECs was measured by capillary tube formation assay. ^▲▲▲^*P* < 0.001, ^▲▲^*P* < 0.01, ^▲^*P* < 0.05.

To explore the molecular mechanisms of how Twist promotes angiogenesis in pancreatic cancer. Using several well-developed miRNA algorithms, such as PicTar, TargetScan and miRNA.org, we found that Twist might be a potential target of miR-497 (Figure 5A). qRT-PCR analysis revealed that the expression of endogenous mi-497 was significantly increased in LV-shTwist-infected Bxpc-3 cells but decreased in LV-Twist-infected Capan-1 cells (Figure 5B). Next, dual-luciferase reporter assay was used to unveil whether miR-497 regulate the expression of Twist directly or indirectly. The relative luciferase activity in the miR-497 mimic co-transfection of vectors that contained the Twist-3′UTR-wt group was markedly decreased compared with control (Figure 5C, left). On the contrary, such activity was increased in miR-497 depleted Bxpc-3 cells (Figure 5C, right). However, the regulatory effect of miR-497 on luciferase activity was abolished following the co-transfection of Twist-3′UTR-mu in Bxpc-3 cells (Figure 5C). In addition, the expression of Twist and VEGFA were significantly increased in anti-miR-497 transfected cells in comparison with those transfected with anti-control, and the ectopic expression of miR-497 in Bxpc-3 cells decreased the protein level of Twist and VEGFA (Figure 5D). These data indicated that Twist is a direct target of miR-497.

### The function role of miR-497 on the cell growth, invasion and tubule formation of HUVECs in *vitro*

Our study demonstrated that the down-regulation of Twist can inhibit metastasis and angiogenesis in pancreatic cancer, along with the confirmation of Twist being a direct target of miR-497. However, it is currently obscure whether miR-497 involves in pancreatic cancer cell angiogenesis. To elucidate whether miR-497 could suppress the angiogenic activity of pancreatic cancer cells. MiR-497 mimic, anti-miR-497 and their matched controls were transfected into Bxpc-3 and Capan-1 cells, and explored the effect of miR-497 on the biological behaviors of HUVECs in *vitro*. As shown in Figure 5E, the cell growth of HUVECs was significantly inhibited after transfection with miR-497 mimic at the 48 h, 72 h and 96 h. The CM from miR-497-upregulated Bxpc-3 cells exhibited fewer invaded cells and tubule formation than the control group (Figure 4F-4G). Whereas the down-regulated of miR-497 promoted cell growth, invasion and tubule formation of HUVECs (Figure 4E-4G). Our results showed that the over-expression of miR-497 inhibits angiogenic activity of HUVECs, resembling the effects of Twist knockdown on HUVECs.

### Twist promotes pancreatic cancer angiogenesis through miR-497 targeting of VEGFA

**Figure 6:**
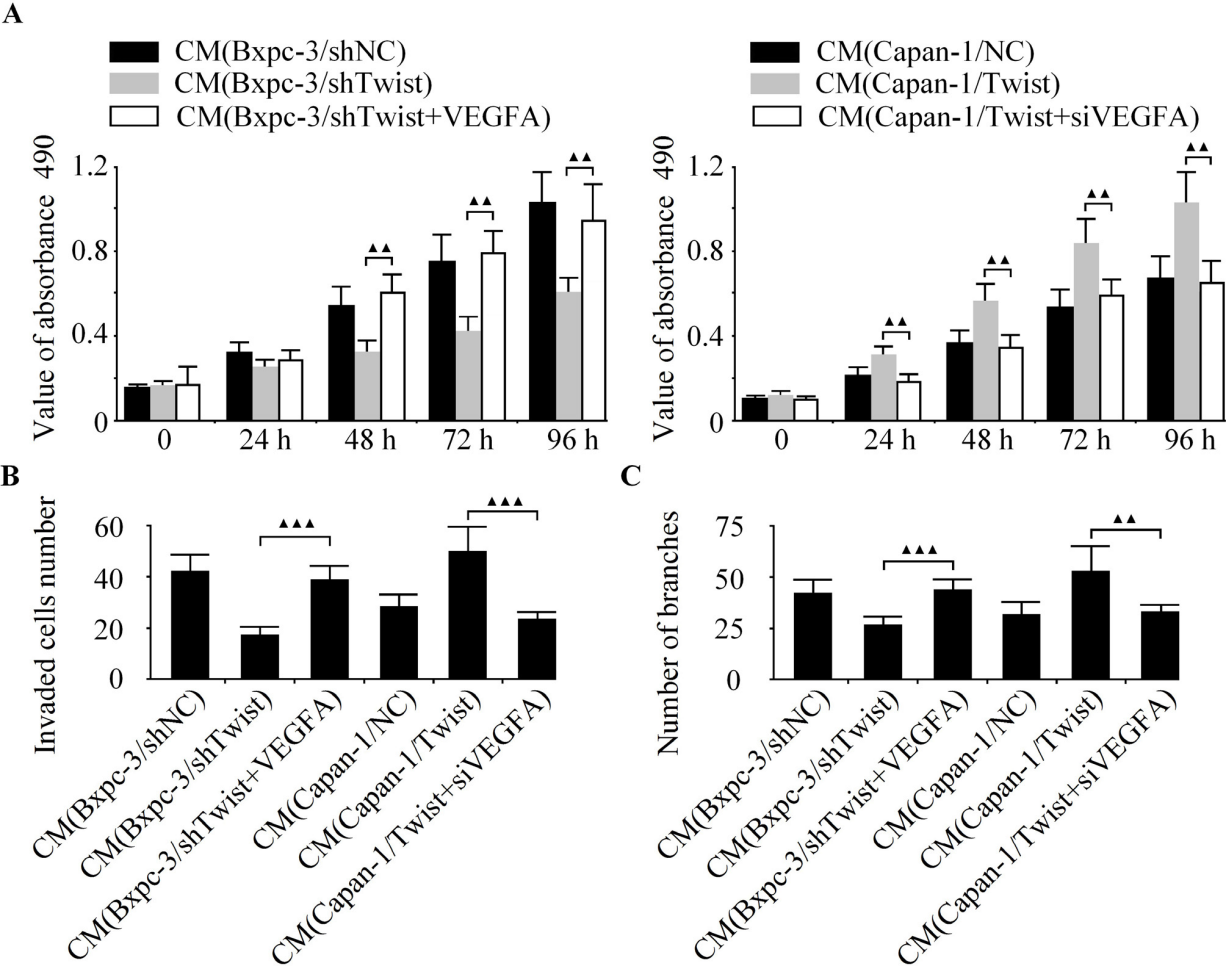
Twist promotes pancreatic cancer angiogenesis through miR-497 targeting of VEGFA Twist-depleted Bxpc-3 cells were pre-incubated with VEGFA over-expressing vector. LV-Twist infected Capan-1 cells were transfected with siVEGFA. Then the media were collected as CM and applied to HUVECs. The cell growth, invasion and tubule formation of HUVECs were detected by CCK-8 **A.** boyden chamber **B.** and capillary tube formation assay **C.** ^▲▲^*P* < 0.01, ^▲^*P* < 0.05.

As a novel promoter of tumor angiogenesis, VEGFA was reported to be a direct target of miR-497 in liver cancer [18]. Nonetheless, how VEGFA related to angiogenesis in pancreatic cancer remains unknown. To investigate whether VEGFA contributed to Twist-induced angiogenesis in pancreatic cancer, we successfully acquired VEGFA-overexpressed Bxpc-3 and VEGFA-depleted Capan-1 cells. We found that the ectopic expression of VEGFA obviously abrogated the anti-angiogenic effect induced by Twist knockdown, and the silencing of VEGFA markedly rescued the pro-angiogenic effect of Twist (Figure 6A-6C). The hypothesis derived was that Twist-induced angiogenesis in pancreatic cancer was correlated by VEGFA.

### The level of twist is positively correlated with VEGFA expression in pancreatic cancer specimens

**Figure 7:**
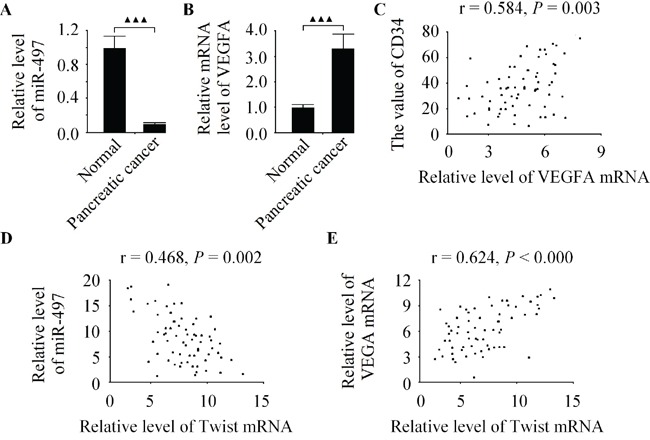
The level of Twist is positively correlated with VEGFA expression in pancreatic cancer specimens **A.** Relative expression of miR-497 in 68 pancreatic cancer tissues was inhibted in comparison to matched adjacent normal pancreatic tissues. **B.** The level of VEGFA was significantly higher in pancreatic cancer specimens than in normal pancreatic specimens. **C.** VEGFA mRNA expression was positively correlated with MVD in pancreatic cancer tissues. **D.** The level of Twist was inversely correlated with miR-497 expression in pancreatic cancer tissues. **E.** A positive correlation between Twist and VEGFA levels in pancreatic cancer tissues. Data are expressed as the mean ± SD, (n = 68), ^▲▲▲^*P* < 0.001.

To examine the putative relationship between Twist and VEGFA in pancreatic cancer tissues. The expression levels of miR-497 and VEGFA in 68 cases of pancreatic cancer specimens as well as their matched adjacent normal pancreatic tissues were analyzed by qRT-PCR. As shown in Figure 7A-7B, in comparison with normal tissues, the levels of miR-497 and VEGFA mRNA were down-regulated and up-regulated in pancreatic cancer tissues, respectively. Moreover, the VEGFA and MVD in pancreatic cancer tissues were found significantly positive correlated to each other (Figure 7C). Additionally, the expression of miR-497 was inversely correlated with the level of Twist (Figure 7D). A positive correlation between Twist and VEGFA levels in pancreatic cancer specimens was observed in this study (Figure 7E). Investigations into such information reflected that VEGFA was closely associated with tumor angiogenesis in pancreatic cancer tissues.

## DISCUSSION

As a member of a highly conserved transcription factor family, Twist has been implicated in the invasion and migration of several solid tumors [14-16]. Recent study demonstrated that the role of Twist in tumor cell invasion and metastasis maybe associated with angiogenesis [17]. However, to the best of our knowledge, there is limited information regarding the molecular mechanisms by which Twist contributes to angiogenesis. This paper highlighted the utilisation of the genetic loss of function and the gain of function techniques to explore the functional role of Twist in tumorigenesis. In this study, we showed for the first time that Twist played an important role as a promoter of angiogenesis in human pancreatic cancer through regulation of miR-497/VEGFA axis.

It is reported that the expression of Twist is decreased in several cancer cell line, and associated with poor prognosis in multiple tumor types [29-32]. Our results showed that the level of Twist is decreased in several pancreatic cells in comparison with normal cells. In addition, a significant increase of Twist expression in pancreatic cancer as well as an association between Twist and a poor prognosis were observed, which also supports the positive regulation effect of Twist on cell malignant biological behaviors. Recently, increasing evidence showed that angiogenesis is essential for the growth of solid tumor and tumor angiogenesis research has become one of the most active fields in tumor treatments [33]. Therefore, identifying anti-angiogenesis targets is considered effective in tumor treatment. In the present study, we found that high level of Twist was positively correlated with MVD in pancreatic cancer tissues. Thus, we hypothesized that Twist may act as a angiogenesis promoter in pancreatic cancer. Functional studies demonstrated that ectopic expression of Twist promoted angiogenesis in *vitro* by stimulating the cell growth, invasion and tubule formation of HUVECs. Further approaches were employed to validate the hypothesis that the effects of Twist on the metastasis and angiogenesis of pancreatic cancer cells are sustained in *vivo*. Using a pancreatic cancer orthotopic xenograft model, we tested the ability of Twist-depleted cells to form angiogenesis and metastatic nodules in nude mice. In *vivo* results demonstrated that the silencing of Twist significantly decreases the vascular density and metastatic nodules of pancreatic cancer. These in *vivo* results are consistent with the changes observed in the cultured cells. Our in *vitro* findings along with in *vivo* results provide us with confidence in the significant value of Twist in the regulation of pancreatic cancer angiogenesis.

Over the past decades, researches on the roles of miRNA in the development of malignant tumors have been a hot topic. MiRNAs are crucial players in many pathophysiological processes owing to their promising potential of being novel diagnostic and predictive markers for therapies. Previous studies demonstrated that several miRs can stimulates the expression of Twist [34-36]. In this study, Twist is identified as a new direct target of miR-497. MiR-497 was reported to inhibit metastasis and angiogenesis in breast [18], ovarian [37] and lung [38] cancer. However, the relationship between miR-497 and angiogenesis has not yet been investigated in pancreatic cancer. Our results found that miR-497 was decreased in pancreatic cancer tissues, which was in consistent with the published reports [18, 39]. Moreover, functional studies demonstrated that the over-expression of Twist could inhibit the level of miR-497 in pancreatic cancer cells, and the expression of miR-497 was markedly increased in Twist-depleted Bxpc-3 cells. In addition, there is a significant inverse correlation between miR-497 and Twist in pancreatic cancer specimens. The over-expression of miR-497 suppresses the angiogenesis in *vitro*, consistent with the effects of Twist knockdown in the same cells. Together, our data suggesting that miR-497 exerted as a anti-angiogenic factor in pancreatic caner cells. So Twist might be a candidate therapeutic target for pancreatic cancer patients and an effective biomarker to predict survival.

VEGFA is frequently over-expressed in a wide range of human tumors [18, 28]. Tumor cell-produced VEGFA can contribute to tumor cell progression and metastasis through stimulating tumor cell survival, migration and invasion [31]. Nowadays, VEGF antagonists such as Bevacizumab have been used safely in humans alone or in combination with chemotherapy [39]. Consistent with such observations, this paper found that VEGFA was markedly increased in pancreatic cancer tissues, and high level of VEGFA was closely associated with tumor angiogenesis in pancreatic cancer. Furthermore, the anti-angiogenic effect of Twist knockdown was almost completely abrogated by the raised expression of VEGFA, whereas the pro-angiogenic effect of Twist was markedly rescued by the silencing of VEGFA. Our results conclusively demonstrated that the angiogenic effect of Twist is correlated by VEGFA in pancreatic cancer angiogenesis.

### Conclusion

In conclusion, our current findings are consistent with the hypothesis that Twist promotes angiogenesis via miR-497/VEGFA axis in pancreatic cancer. Moreover, Twist was identified as a direct physiological target of miR-497. This work provide further evidence for the role of the Twist in the functional regulation of pancreatic cancer. In this way, Twist can become a novel therapeutic target for pancreatic cancer treatment in the future.

### Open access

This article is distributed under the terms of the Creative Commons Attribution License which permits any use, distribution, and reproduction in any medium, provided the original author(s) and the source are credited.

### Ethical statement

The ethic committee of Nanfang Hospital and The First People Hospital of Yueyang had approved this trial in October, 2003. Informed consent was obtained from all individual participants included in this study. This clinical trial had been registered on Clinical Trials.gov (NCT01334562).

## MATERIALS AND METHODS

### Cell culture

Human pancreatic cancer cell lines Bxpc-3, MIA PaCa-2, Capan-1, Panc-1 and HPAC as well as HUVECs were purchased from American Type Culture Collection (ATCC, Rockville, MD). Normal human pancreatic duct epithelial cells (HPDEC) and HEK293T cells were preserved in our laboratory. Pancreatic cancer cell lines and HEK293T cells were cultured in RPMI-1640 medium (Gibco, NY, USA) containing 10% FBS (Gibco) and 0.1 IU/mL insulin. HUVECs were cultured in endothelial cell basal medium (Gibco). HPDEC were maintained in keratinocyte serumfree medium (Gibco) with EGF (1 ng/mL) and BPE (50 mg/mL). All cell lines were cultured with 100 U/mL penicillin, 100 μg/mL streptomycin and 1% glutamine at 37°C with 5% CO_2_ atmosphere.

### Patients and tissue specimens

68 human pancreatic cancer clinical samples and their corresponding normal pancreatic tissues were obtained from pancreatic cancer patients who underwent operation of pancreatic cancer at Nanfang Hospital and The First People Hospital of Yueyang between December 2003 and December 2008. All cases were histologically confirmed by trained pathologists. No patients received chemotherapy or radiotherapy prior to surgery. The patients’ profiles are summarized in Table 1. All the specimens were obtained with informed consent and approved by the ethics committee of Nanfang Hospital and The First People Hospital of Yueyang.

**Table 1:** The clinicopathological characteristics of 68 patients

Feature	No. of patients
Overall	68
Age (yrs)	
< 65	24
≥65	44
Gender	
Male	35
Female	33
Grage	
well differentiated	34
moderately differentiated	26
poorly differentiated	7
undifferentiated	1
Clinical stage	
I	6
II	18
III	23
IV	21
Twist expression	
Twist negative	11
Twist +	24
Twist ++	33

### RNA transfection, plasmid construction and lentivius transduction

The recombined lentiviruses LV-Twist and LV-shTwist were constructed in Capan-1 and Bxpc-3 cells respectively. The empty lentiviral vector LV-control was used as a negative control (NC). The production, purification and titration of recombinant lentivirus were prepared as previously descried [25]. MiR-497 mimic, inhibitor (anti-miR-497) and their matched negative controls (mimic control or anti-control) were synthesized by GenePharma (Shanghai, China). VEGFA over-expressing vector, VEGFA gene-specific short interfering (siRNA) and non-specific control siRNA were purchased from Clontech Laboratories, Inc. The transfection was conducted using lipofectamine 2000 (Invitrogen, CA, USA) according to manufacturer's construction. Complete medium was changed 5 h after transfection. Double-stranded oligonucleotides corresponding to the wild-type (wt) or mutant (mu) miR-497 binding site in the 3′UTR of Twist (Twist-3′UTR-wt and Twist-3′UTR-mu) were synthesized into the pMIR-REPORT system (Applied Biosystems).

### Quantitative reverse transcription polymerase chain reaction (qRT-PCR)

Total RNA extraction was performed using Trizol Reagent (Life Technologies, Carlsbad, CA, USA) according to the manufacturer's instructions. cDNA was produced according to the protocol for PrimeScript™ RT Reagent (TaKaRa, Japan). To measure the mRNA levels of Twist and VEGFA as well as miR-497, total RNA was analyzed using Bulge-LoopTM miRNA qRT-PCR Primer (Applied Biosystems, Foster City, CA) and normalized to U6. Each reverse transcript was amplified with GAPDH as an internal control. The relative expression was calculated using the 2^ΔΔCt^ method [7]. All experiments were performed in triplicate.

### Western blot analysis

Protein was extracted using Mammalian Protein Extraction Reagent (Pierce Inc., Rockford, IL) and its concentration was determined by BCA (Pierce, Rockford, IL, USA) assay. Total proteins (20~40 μg) from each sample were electrophoresised on 8 % SDS-PAGE gel, and transferred to a nitrocellulose membrane. The membranes were blocked in 5% nonfat milk and probed with the primary antibodies to mouse monoclonal Twist (Abcam, Cambridge, MA, USA) and goat polyclonal VEGFA (Sigma, St Louis, Mo, USA) as indicated overnight at 4°C, and then with the respective secondary antibodies. Band signals were visualized using an enhanced chemiluminescence kit (Pierce, Minneapolis, MN, USA). The same membrane was reprobed with the anti-β-actin antibody, which was used as the internal control.

### Preparation of tumor cell conditioned medium (CM)

After pancreatic cancer cells were transfected for 48 h, then the supernatant medium was replaced by serum free medium and incubation for another 24 h. The CM were collected after centrifugation at 4°C, 2,000 rpm for 10 min and stored at -70°C for subsequent use.

### CCK-8 assay

HUVECs were seeded in each well of 96-well culture plates (1000 cells per well). After overnight incubation, medium was removed and replaced with fresh culture medium plus equal amounts of different CM. After 48 h incubation, the supernatant was discarded and 10 μL CCK-8 solution (Dojin Laboratory, Kumamoto, Japan) was added, then the cells were incubated at 37°C for 60 min, the absorbance was measured at 490 nm using a microplate spectrophotometer (Bio-Tek, USA). This experiment was repeated twice.

### Cell invasion assay

The invasiveness of HUVECs in *vitro* was assessed by BD Matrigel^®^ invasion chamber. Briefly, cells (2 × 10^5^/mL) in 0.5 mL serum-free medium were placed in the upper chamber, and the lower chamber was filled with 0.6 mL of medium with 20% FBS. After 24 h incubation, the lower surfaces of the membrane were fixed with 100% methanol and stained with crystal violet. Cells that had invaded to the lower surface of the membranes were counted under a microscope in five predetermined field.

### Capillary tube formation assay

For tubule formation Assay, matrigel (Becton Dickinson, USA) was dissolved at 4°C for overnight, and each well of prechilled 96-well plates was coated with 100 μL matrigel and incubated at 37°C for 45 min. HUVECs were transferred to 96-well plates by different CM at the density of 1 × 10^4^/well for 12 h in a humidified 5% CO_2_ atmosphere. Capillary-like structures of HUVECs were photographed and the light micrograph images were stored in a computer. Tubular structures were quantified by manual counting at the 100 × magnification.

### Dual luciferase reporter assay

Luciferase assay was carried out on extracts from different pancreatic cancer cells co-transfected with Twist-3′UTR reporter plasmids and miR-497 mimic or mimic control for 24 h using a dual luciferase reporter assay kit (Promega, Madison, WI, USA) according to the manufacturer's recommendations. The firefly luciferase (FL) reporter was measured by a microplate spectrophotometer. Renilla luciferase (RL) activity was normalized to FL activity. All experiments were performed three times.

### Orthotopic implantation of pancreatic cancer

In *vivo* experiments were conducted as described previously [26]. Briefly, we used male athymic BALB/c nu/nu mice (4~6 weeks old) that have been maintained in the standard mice plexiglass cages in a room maintained at constant temperature and humidity under 12 h light and darkness cycle. Briefly, 1 × 10^7^ logarithmically growing Bxpc-3/shNC or Bxpc-3/shTwist cells were subcutaneously injected into the mid-abdominal of nude mice. After the tumors had reached a size of 5 mm^3^, the mice were sacrificed and the subcutaneous tumors were surgically excised, necrotic tissues were cut away and the remaining healthy tumor tissues were minced into approximately 2×2×2 mm pieces macroscopically.

Forty nude mice were anesthetized with pelltobarbitalum natricum, then a longitudinal incision was made through the left abdominal pararectal. The pancreatic tail was carefully exteriorized, and a tumor tissue piece was gently transplanted into the pancreas parenchyma with a 6-0 surgical suture. Then relocation the pancreas and spleen into the abdominal cavity, and the muscular layer and the skin were closed with 6-0 surgical suture respectively. The survival of nude mice was evaluated up to day 120. At the termination of experiment, primary pancreatic tumor and the number of macroscopic nodules on the peritoneal surface or intestine were evaluated respectively.

### Tumor vascular imaging

Tumor vascular imaging was conducted using the lead oxide-gelatin injection technique. Based on the same orthotopic transplantation model as above. The tumor-bearing nude mice were anesthetized with pelltobarbitalum natricum on day 40. After a 1.0 cm incision was made in the middle of the chest, and the ribs were severed to completely expose the heart. Then, a catheter was inserted into the aortic arch from heart apex. The lead oxide injectate was perfused through the catheters at the dose of 20 mL/kg. After the nude mice were stored at -20°C overnight, the primary pancreatic tumors were excised neatly. In the end, the vascular images of the primary tumors were captured using an X-ray machine. Scion Image Beta 4.02 software (Scion Corporation, Frederick, MD, USA) was used to analyzed the blood vessel volume in the images. The vascular density was quantified as the total blood vessel volume in tumor divided by the tumor volume.

### Immunohistochemical and microvessel density (MVD) evaluation

Tumor samples were embedded in paraffin and fixed with paraformaldehyde. After being washed in PBS, the slides were blocked with protein block solution (DakoCytomation) to obstruct endogenous peroxidase activity. Such samples were then incubated overnight with specific antibody against CD34 (Sigma), Twist or VEGFA with appropriate dilution. Normal host serum was used for negative control, followed by staining with appropriate HRP-conjugated secondary antibody. The peroxidase was visualized with 3-3′-diaminobenzidinetetrahydrochloride solution and then counterstained with a weak hematoxylin solution stain. The stained slides were dehydrated and visualized on a microscope (Olympus, Japan) by an investigator that was blinded to the xenograft tumor groups. The stained sections were counted in 10 random views at 400 × magnification. In each view, the grayscale intensity was measured at 20 randomly selected points and averaged. The stained sections were counted in the five areas of highest vascular density at 400 × magnification and the MVD was expressed as the mean number of vessels in these areas, and the value of integrated option density was proportional to the Twist or VEGFA protein level.

### Statistical analysis

Data were represented as mean ± SD for the absolute values or percent of controls. Statistical differences were evaluated by one-way analysis of variance (ANOVA) followed by dunnett-tmultiple comparisons tests. The relations between CD34 and Twist or VEGFA as well as the relationships between Twist and miR-497 or VEGFA were analyzed by correlation analysis. Survival analysis was evaluated by Kaplan-Meier survival plot followed by a log-rank test. All calculations were performed with the SPSS software (version 17.0, SPSS, Inc.,). A value of *P* < 0.05 was considered statistically significant.

## Abbreviations

VEGFA: vascular endothelial growth factor a
HUVEC: human umbilical vein endothelial cells
HPDEC: Normal human pancreatic duct epithelial cells
LV: lentivirus; siRNA: short interfering
sh: short hairpin
wt: wild-type
mu: mutant
miRNAs: microRNAs
NC: negative control
UTRs: untranslated regions
qRT-PCR: quantitative reverse transcription polymerase chain reaction
CM: conditioned medium
CCK-8: cell counting kit-8
MVD: microvessel density
